# Yaws in the Philippines: first reported cases since the 1970s

**DOI:** 10.1186/s40249-019-0617-6

**Published:** 2020-01-30

**Authors:** Belen Lardizabal Dofitas, Sherjan P. Kalim, Camille B. Toledo, Jan Hendrik Richardus

**Affiliations:** 1 Philippine Leprosy Mission, Inc., University of the Philippines Manila - College of Medicine, Manila, the Philippines; 2 Department of Public Health, Erasmus MC, University Medical Center Rotterdam, Rotterdam, The Netherlands; 3 Cotabato Regional and Medical Center – Department of Pathology, Cotabato City, the Philippines; 4 Rural Health Unit, Municipality of Tulunan, North Cotabato province & Southern Philippines Medical Center, Davao City, the Philippines

**Keywords:** Yaws, Neglected tropical diseases, The Philippines

## Abstract

**Background:**

Yaws is a chronic, highly contagious skin and bone infection affecting children living in impoverished, remote communities and caused by *Treponema pallidum* subspecies *pertenue*. The Philippines was thought to be free of yaws following the 1950s eradication campaign but it has been reported in the Liguasan Marsh area, Central Mindanao. This is the first documentation of yaws cases in the Philippines since the 1970s. We describe active and latent yaws recently detected in the Southern Philippines.

**Case presentation:**

Cross-sectional surveys and screening of skin diseases were conducted in one randomly selected public elementary school per selected municipality in Liguasan Marsh, covering three municipalities per province. Yaws suspects underwent screening and confirmatory serologic tests for *Treponema pallidum* using Dual Path Platform Syphilis Screen and Confirm Assay (DPP) and *Treponema pallidum* Particle Agglutination (TPPA). Children with yaws skin lesions and reactive confirmatory tests for *T. pallidum* and non-treponemal antibodies were considered confirmed yaws cases. Four children aged 5–10 years old had confirmed secondary yaws in Tulunan Municipality, Cotabato Province and in Lambayong Municipality, Sultan Kudarat Province. All had secondary yaws lesions such as moist, cauliflower-like papillomas, thick yellow crusts on pink papules and nodules, whitish, papulosquamous papules and plaques, or hypopigmented patches with small papules on the periphery. Yaws papillomas and erosions were also found on the soles of the feet of one child. The index case had a skin punch biopsy of a partially treated papilloma on his axilla. Histopathological findings showed lichenoid psoriasiform dermatitis with plasma cells, consistent with yaws.

**Conclusions:**

The clinical, serological, and histopathological confirmation of four yaws cases among children has made the Philippines the 14th country endemic for yaws. This report can help health personnel recognize hidden cases of yaws based on skin signs and serological tests. Yaws remained unrecognized and unreported in the Philippines and in countries previously endemic for yaws probably due to the unsustained integration of the yaws program in the general health services and complacency after the 1950s eradication campaign. Our findings have provided the necessary evidence and stimulus to develop a yaws control and eradication program as one of the country’s neglected tropical diseases.

## Background

Yaws is a chronic, contagious, nonvenereal, treponemal infection in humans. Infection with *Treponema pallidum* subspecies *pertenue* causes the disease. Yaws occurs primarily in warm, humid, tropical areas with heavy rainfall, and among poor rural populations where conditions of overcrowding, poor sanitation and inadequate water supply prevail [1]. The disease is known by many different names; *pian* in French, *frambesia* in German, and *bouba* in Spanish. In Malay, it is also called *parangi* and *paru* [2].

Yaws skin lesions are typically thick, dry or moist, yellow-crusted papillomas evolving into ulcers. Transmission is through skin-to-skin contact with fluids from yaws skin lesions, especially when there are breaks in the skin. Majority of active yaws occurs in children under 15 years of age [3].

In 2012, the World Health Organization (WHO) included yaws as one of the neglected tropical diseases targeted for eradication by 2020. Eradication was deemed feasible with the evidence of one dose oral azithromycin as an effective and safe treatment for yaws among children and adults. WHO developed the yaws eradication strategy, also known as the Morges strategy, which recommends Total Community Treatment or Total Targeted Treatment with one dose of oral azithromycin [4].

In the Philippines, yaws was generally thought to have been eliminated after the 1950s nationwide campaign. However, suspected yaws cases were reported in the Liguasan Marsh area, Central Mindanao, prompting the Department of Health to conduct a study to assess the presence or absence of yaws in the country. This report describes the first confirmed yaws cases among Filipinos many years after official reports ceased in 1973.

## Case presentation

In order to confirm the presence of yaws in the Southern Philippines, a clinico-seroprevalence survey commissioned by the Department of Health was conducted from February to May 2017 in three provinces of Mindanao where yaws reportedly continued to exist: Maguindanao, Cotabato, and Sultan Kudarat. Three municipalities per province situated in Liguasan Marsh were purposively chosen based on previous yaws reports. School-based surveys were conducted in one randomly selected public elementary school per selected municipality, totaling nine schools. The study was approved by the St. Cabrini Medical Center-Asian Eye Institute Ethics Review Committee, Manila. The clinico-seroprevalence survey findings are to be published in a separate article (Dofitas B. Yaws in the Philippines: a clinico-seroprevalence study in Mindanao. in preparation).

Preceding the surveys, the investigator conducted orientations about the study and yaws for key stakeholders, local health personnel and school nurses in the municipalities involved. Thereafter, the health personnel conducted orientations for teachers and parents in the selected schools. School teachers instructed the elementary school students to report household members who had any skin disease, guided by flyers with photographs of skin diseases including leprosy and yaws. School nurses pre-screened the students for any pathologic skin lesions especially for yaws. Field teams composed of local health physicians conducted skin examinations of students, household contacts, and community members during scheduled free skin clinic days in the school. Yaws suspects and other patients were referred to the study dermatologist via tele-dermatology.

Blood tests for non-treponemal and treponemal antibodies, namely, Dual Path Platform Syphilis Screen and Confirm Assay (DPP) from Chembio Diagnostic System, Inc., New York, USA and Seriodia®TP-PA *Treponema pallidum* Particle Agglutination (TPPA) from Fujirebio, Inc., Japan, were performed on suspected yaws cases and their household contacts. TPPA was performed in order to determine titers of treponemal antibodies as an additional documentation of the infection.

Children with yaws-like skin lesions and reactive tests for *T. pallidum* and non-treponemal antibodies were considered confirmed active yaws cases while those without yaws-like skin lesions were considered latent yaws cases.

A total of 2779 participants were screened for any skin disease: 2291 students from the selected schools, 393 household members, and 95 community referrals. A total of 2165 (77.91%) were children 15 years old and younger. Blood tests were performed on 150 patients (96 children and 54 adults) with suspected yaws based on skin lesions or as household contacts of yaws cases.

One student was found with active yaws in Sultan Kudarat Province. There were three more confirmed yaws cases among children from a school that was not included in the school study sites and referred to the study’s free skin clinic. These children also resided in one of the selected municipalities of Cotabato Province but they were students of a *madrasa* (a Muslim school) of Maguindanao Province, adjacent to the municipality. The children were brought by the local midwife. The index case was a 10-year old boy (Case 1). A second skin clinic was held a few weeks later at the Rural Health Center so that household contacts and other suspected yaws patients could be checked by a dermatologist, the principal investigator. Two other children (5-year old girl and 9-year old boy) who were not students of the target schools were positive for yaws skin papillomas and were serologically reactive to treponema and non-treponemal antibodies (DPP and TPPA).

Thus, a total of four children (two males, two females) aged 5–10 years were diagnosed as confirmed secondary yaws cases based on typical yaws skin lesions with concomitant serological reactivity to both treponemal and non-treponemal antibodies.

### Description of active yaws cases

#### Case 1

A 10-year-old boy from a selected municipality of Cotabato Province was the first yaws case detected during the study. The index case was referred by health workers after the yaws orientation was given. The skin lesions were of a few months’ duration.

**Fig. 1:**
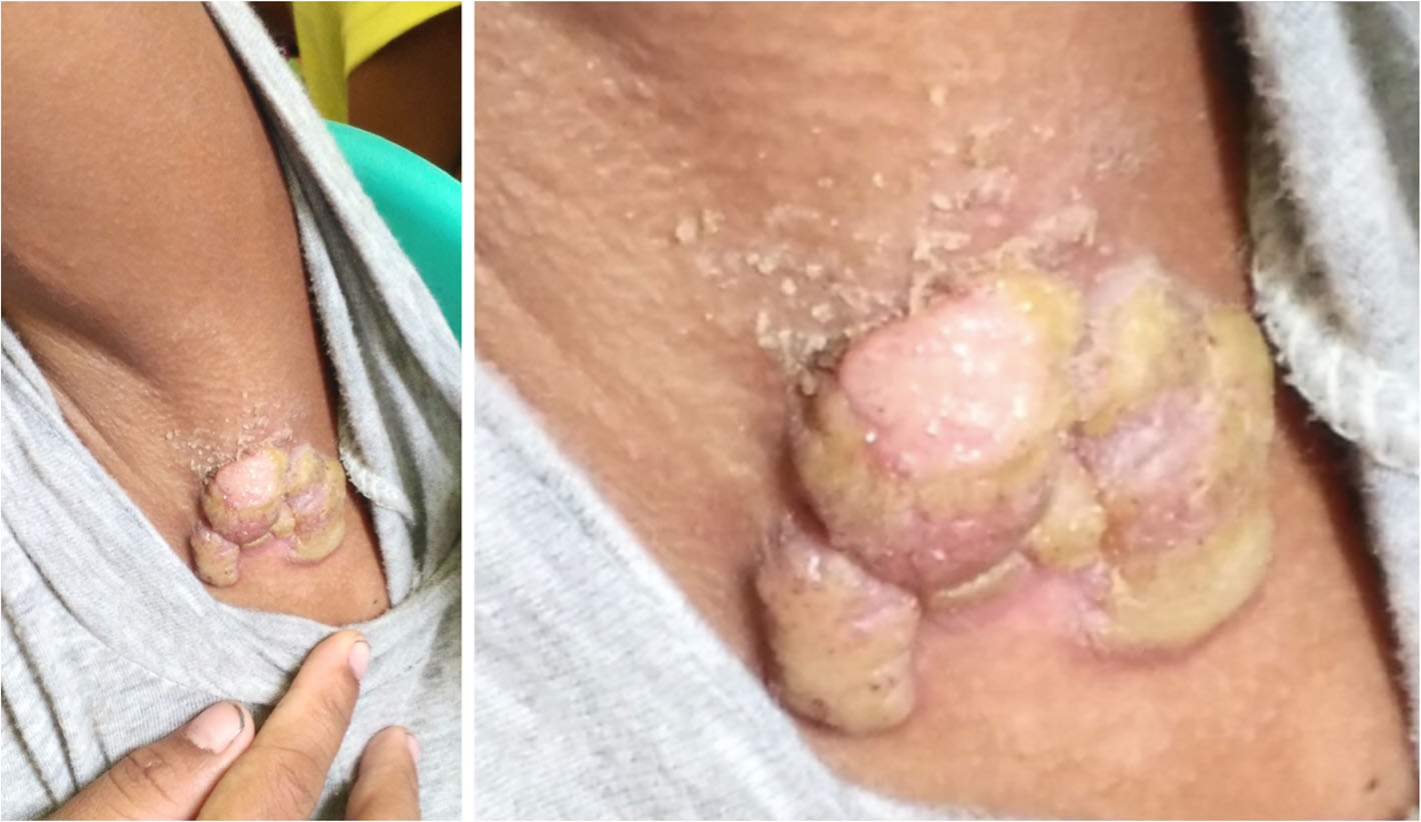
*Case 1: Large, “moist cauliflower” papillomas on left axilla. *Photographs courtesy of Dr. Camille Toledo

**Fig. 2:**
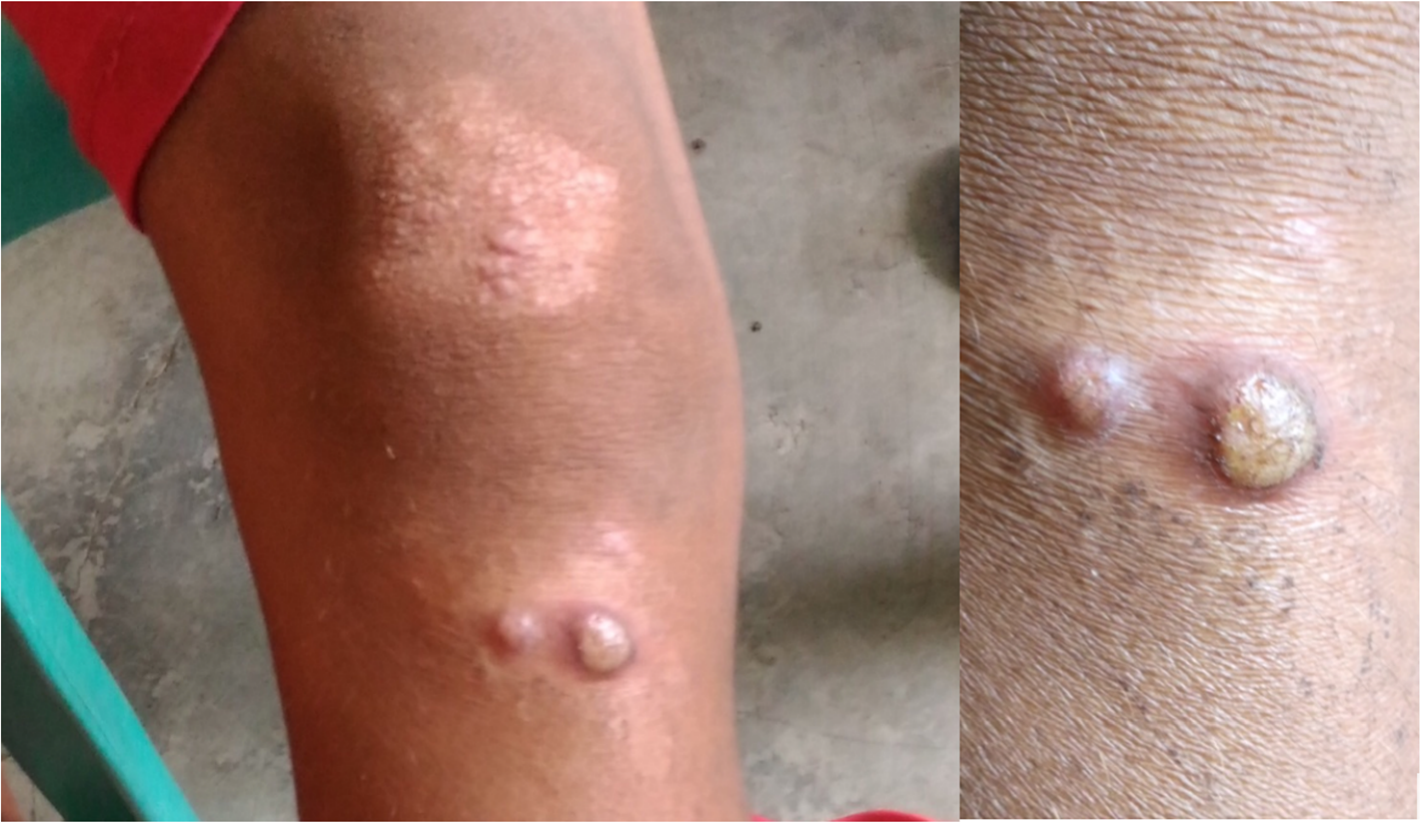
* Case 1: Papulosquamous plaque and yellow-crusted nodules of secondary yaws on the leg (far view and close-up). *Photographs courtesy of Dr. Camille Toledo

Skin findings:

- Large “moist cauliflower” papillomas on the left axilla (Fig. 1)
- Few yellow crusted nodules on the knee (Fig. 2)
- Large, hypopigmented, papulosquamous, irregularly shaped plaque topped with scaly papules on the thigh (Fig. 2)

The mother of the index case was serologically reactive to treponemal and non-treponemal antibodies but without typical yaws skin or bone lesions or syphilis skin lesions. She had no history of syphilis and was assessed to be a latent yaws case. The boy’s younger sister had a history of similar skin lesions but only atrophic scars were noted during skin examination. She refused blood extraction and was assessed to be a yaws suspect.

**Fig. 3:**
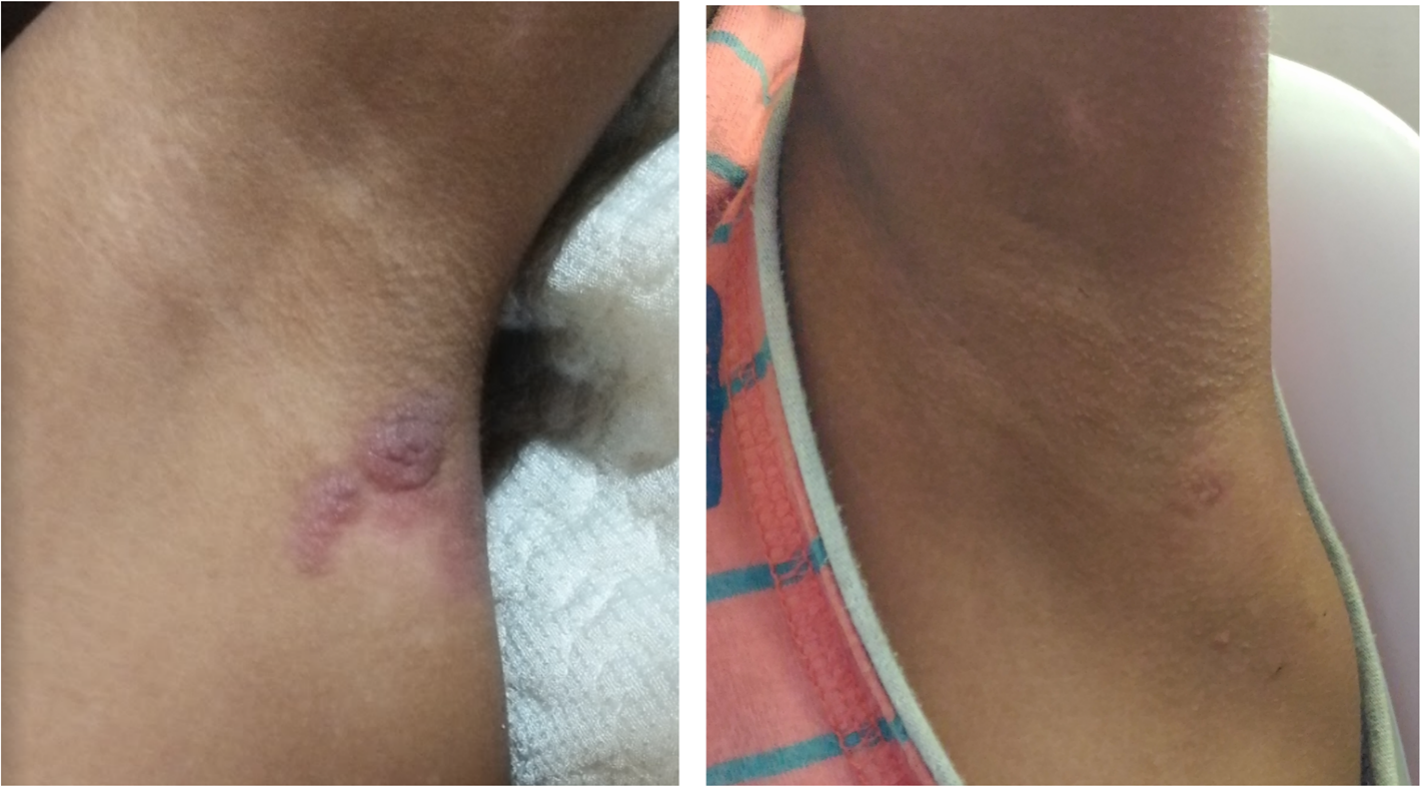
Case 1: Yaws papillomas on axilla significantly reduced in size 2 weeks and completely resolved 3.5 months* after one-dose azithromycin was taken. *Photographs courtesy of Dr. Camille Toledo

**Fig. 4:**
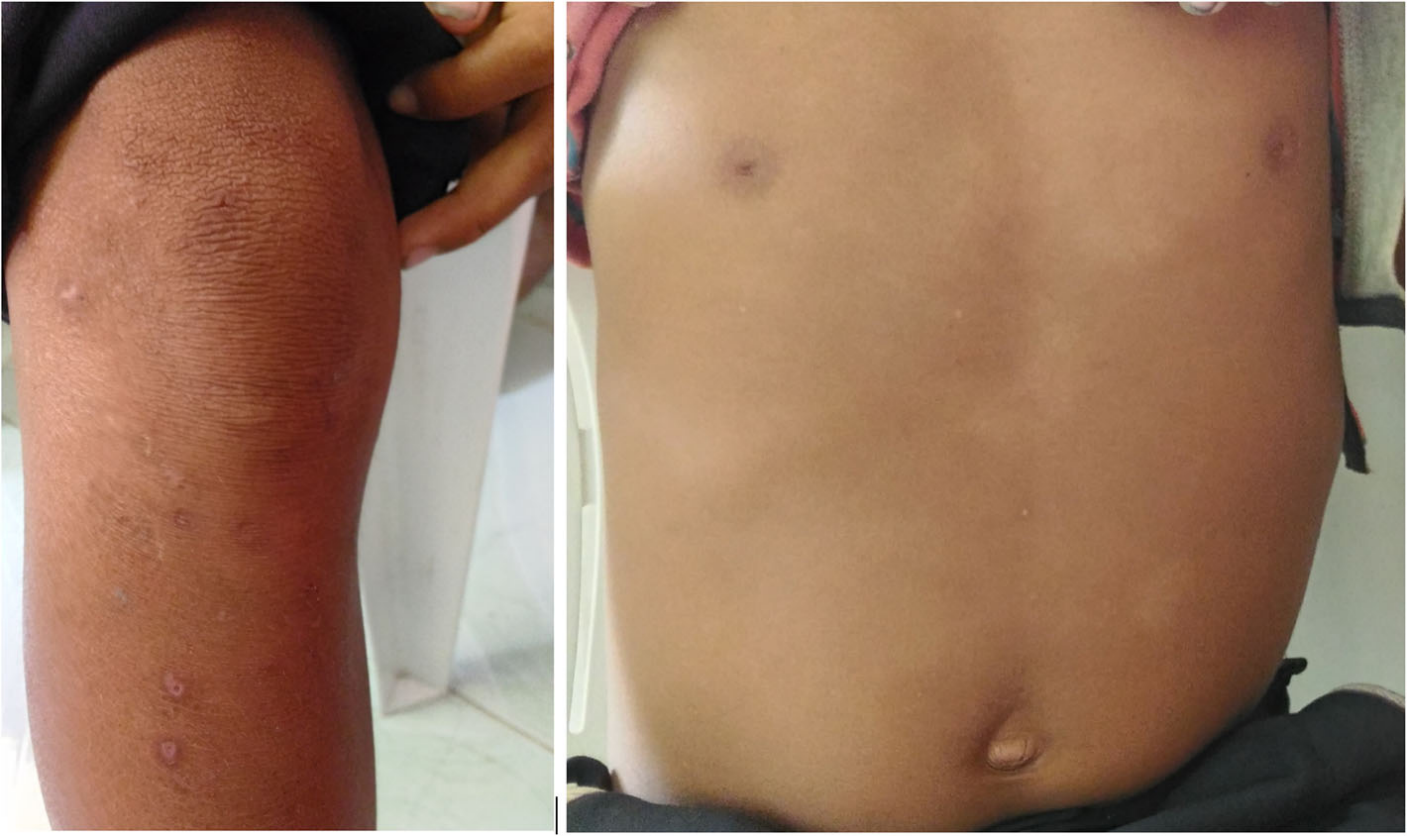
*Case 1: Leg and anterior trunk 3 months post-treatment; Atrophic scars and residual hyperpigmentation and hypopigmenation of resolved yaws lesions after one-dose azithromycin. *Photographs courtesy of Dr. Camille Toledo.

The boy was given one-dose azithromycin (30 mg/kg body weight) and returned for follow-up after 2 weeks and for skin punch biopsy of the axillary papillomas. The yaws lesions were significantly smaller. Full resolution of yaws lesions was noted 3 months later on follow-up by the rural health unit physician (Figs. 3 and 4).

#### Histopathologic findings of yaws papilloma

A skin punch biopsy of the papilloma on the left axilla of the index case (Case 1) was performed by the principal investigator in the Rural Health Center 2 weeks post-treatment. The specimen was processed in the Philippine General Hospital and read by a dermatopathologist of the Division of Dermatology.

**Fig. 5:**
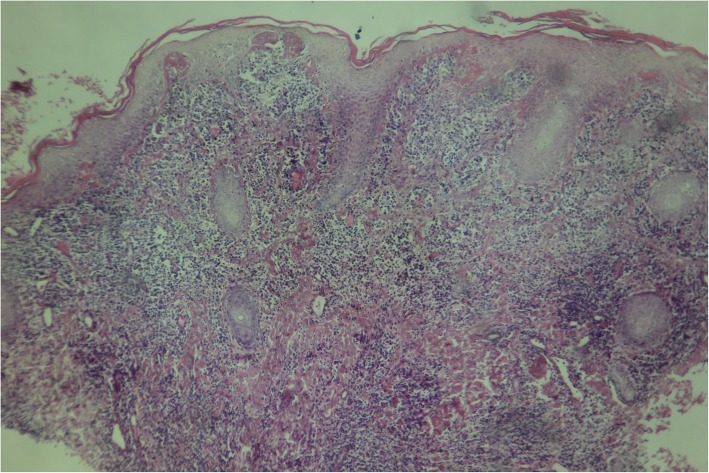
Case 1: (Low power view) Psoriasiform epidermal hyperplasia and band-like lymphocytic infiltrates in papillary dermis

**Fig. 6:**
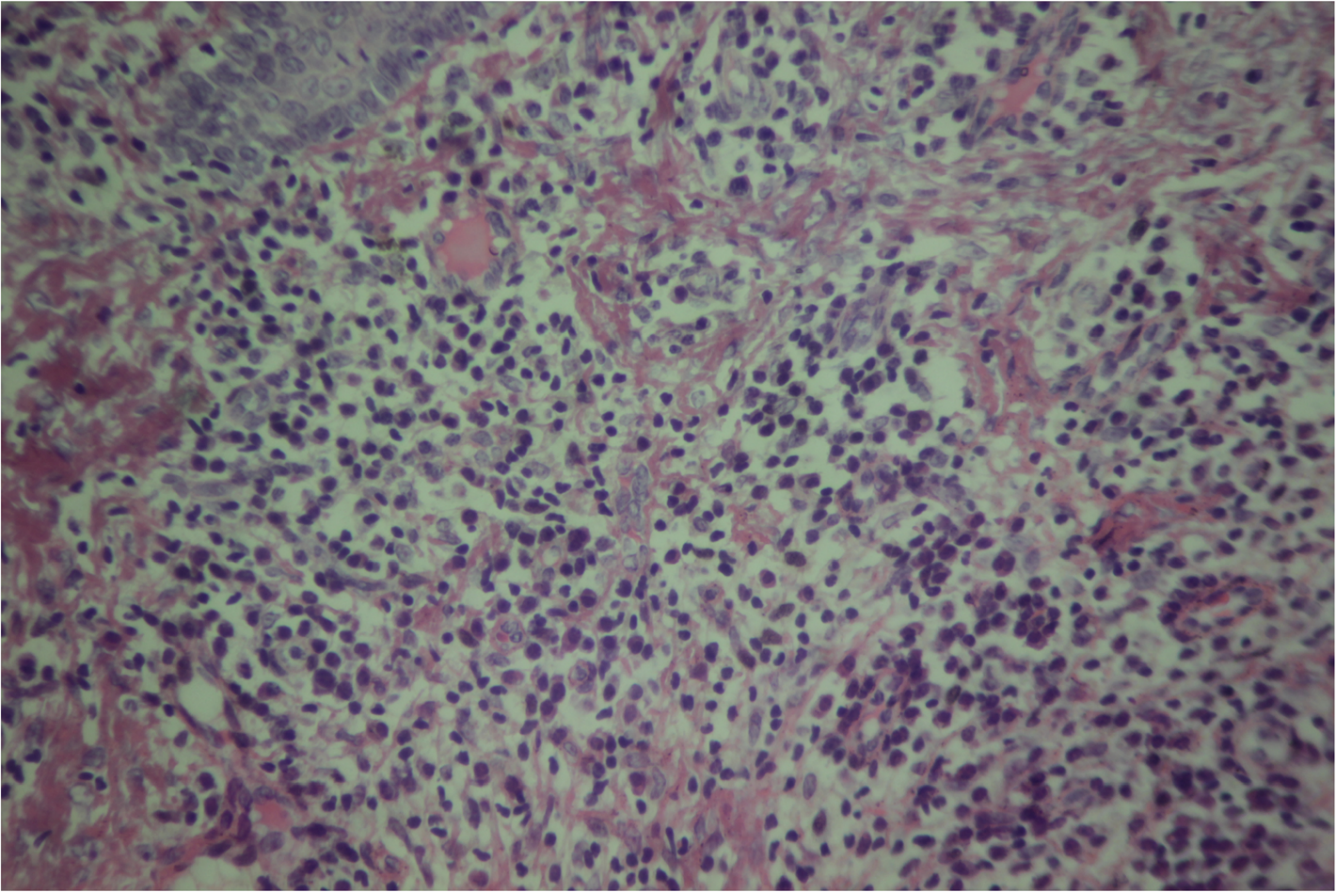
Case 1: (High power view) Moderately dense infiltrate of plasma cells and lymphocytes in the papillary dermis

The biopsy showed psoriasiform epidermal hyperplasia and band-like lymphocytic infiltrates in the papillary dermis, with thickening of the basement membrane. There were moderately dense infiltrates of plasma cells and lymphocytes in the papillary dermis. The histopathologic diagnosis was lichenoid psoriasiform dermatitis with plasma cells. These findings were consistent with yaws (Figs. 5 and 6).

#### Case 2

This concerns a 9-year-old boy from the same village as Case 1. One month prior to the consult, the boy developed multiple pruritic papules on the body and face. Bleeding and pain on the skin lesions were noted.

**Fig. 7:**
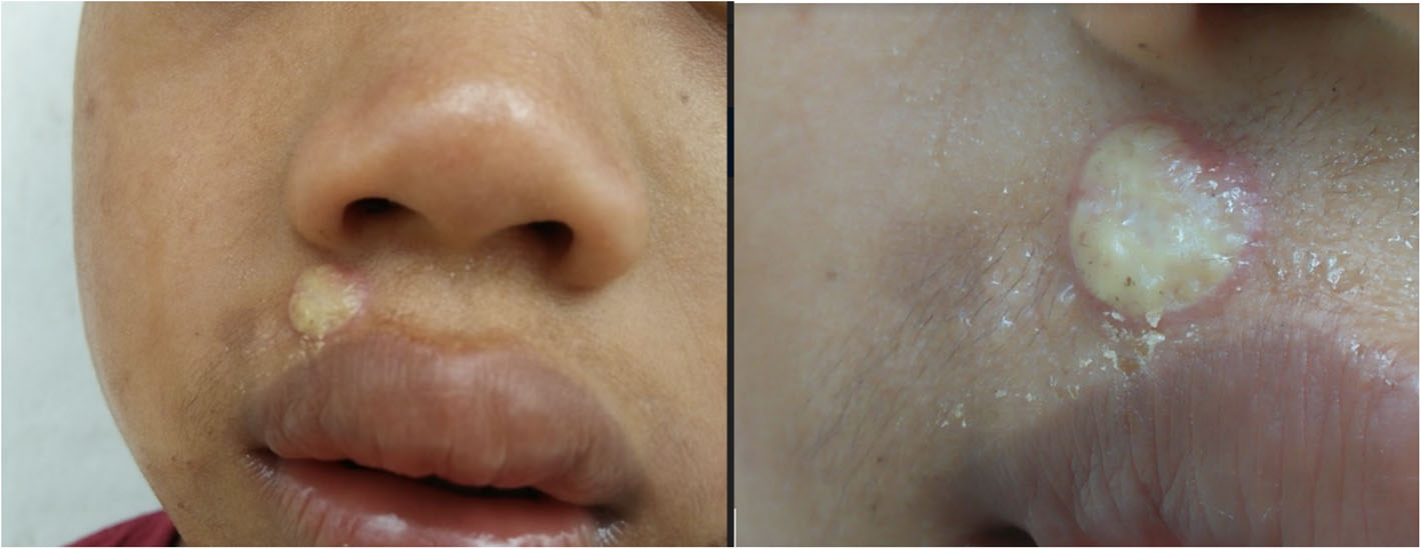
*Case 2: Moist, thick yellow crusted pink papilloma on the face of a nine year old boy (medium and close-up views). *Photographs courtesy of Dr. Camille Toledo.

**Fig. 8:**
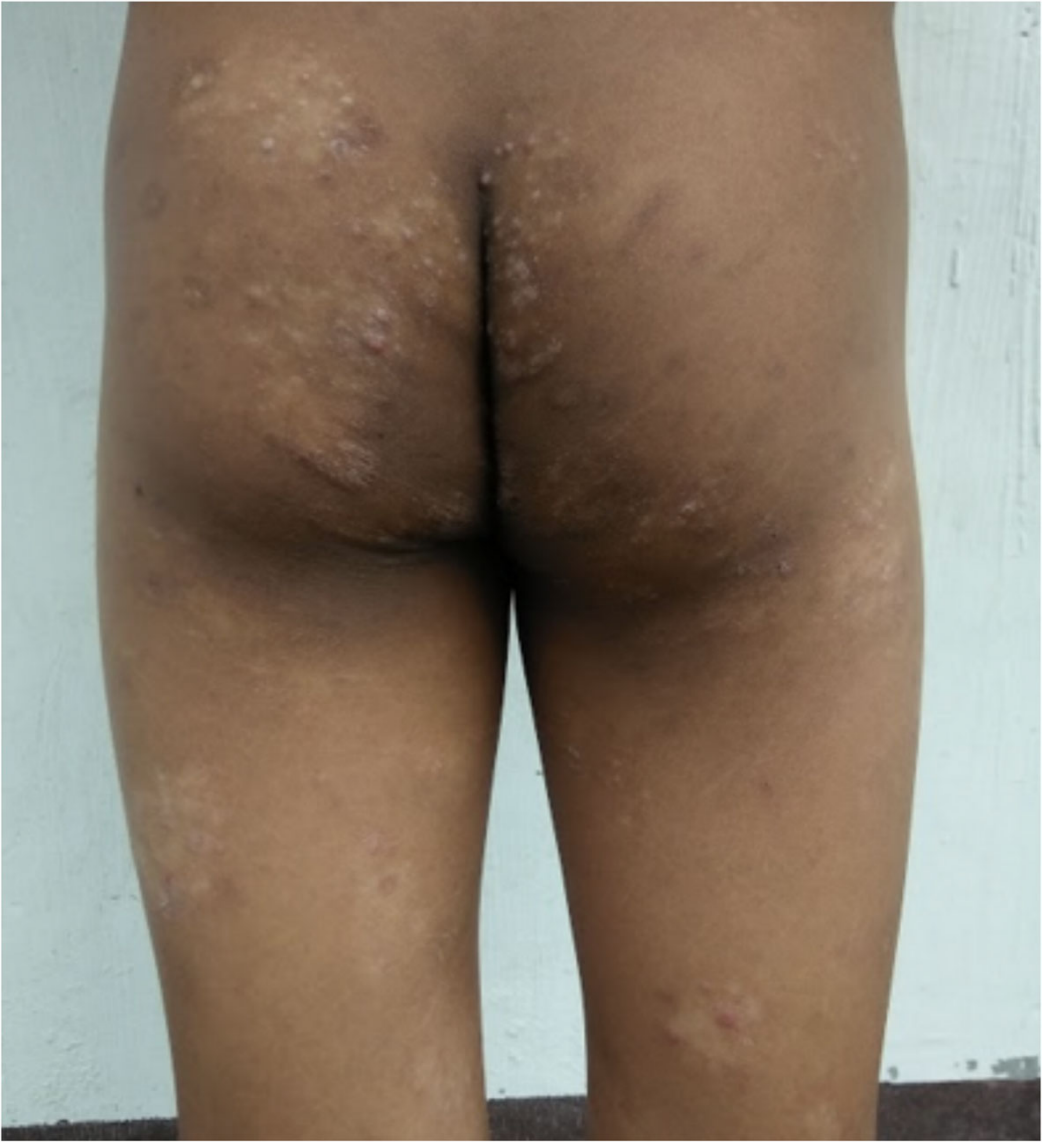
Case 2: Dry, scaly, whitish and flesh-colored papular plaques on the buttocks; hypopigmented irregular patches and macules on thighs and legs

**Fig. 9:**
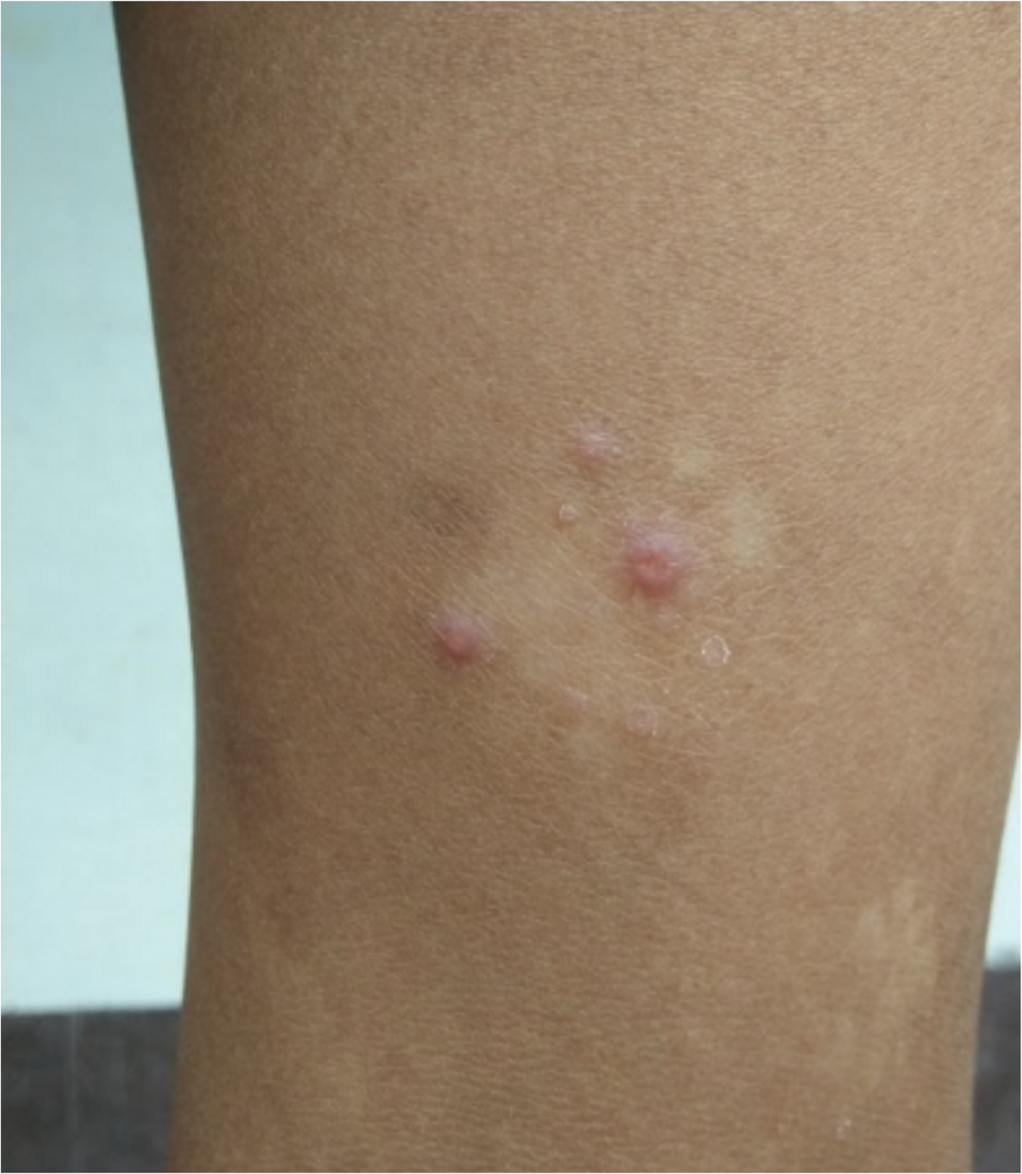
Case 2: Hypopigmented patches with overlying pink papules with central depressions

**Fig. 10:**
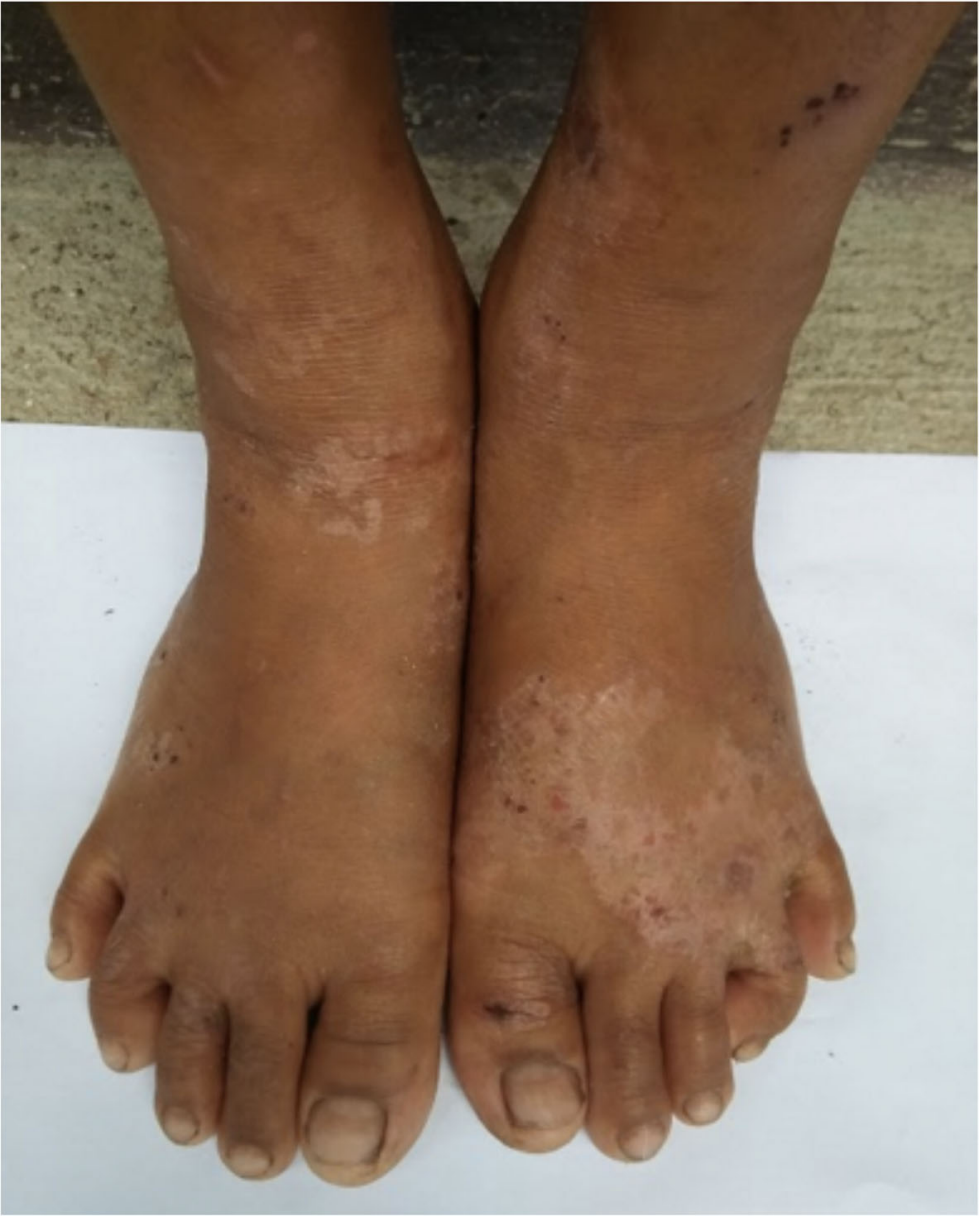
Case 2: Large, hypopigmented patches topped with scaly pink and brown papules on the dorsa of feet and ankles

**Fig. 11:**
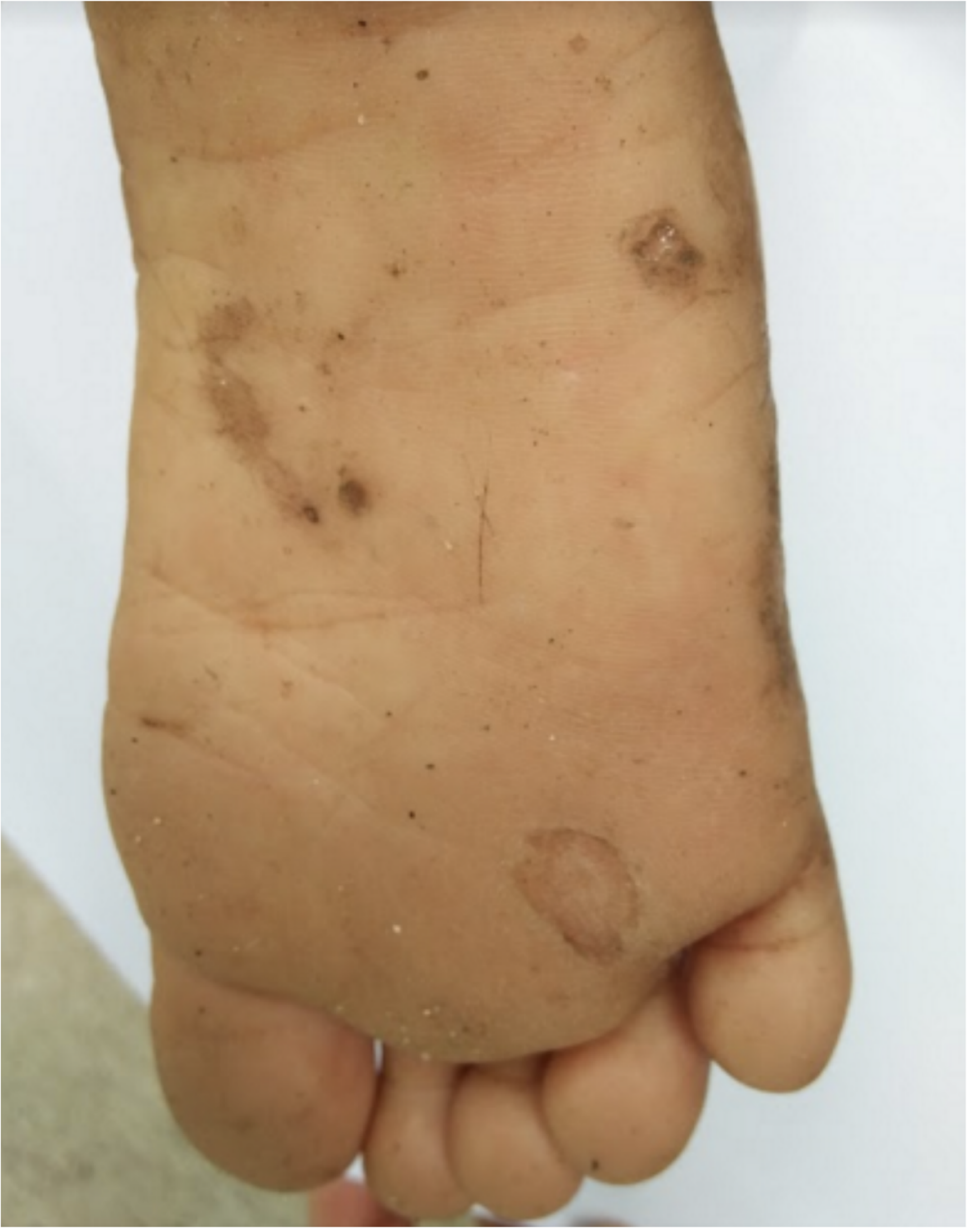
Case 2: Plantar yaws lesions – deep punched-out erosions

**Fig. 12:**
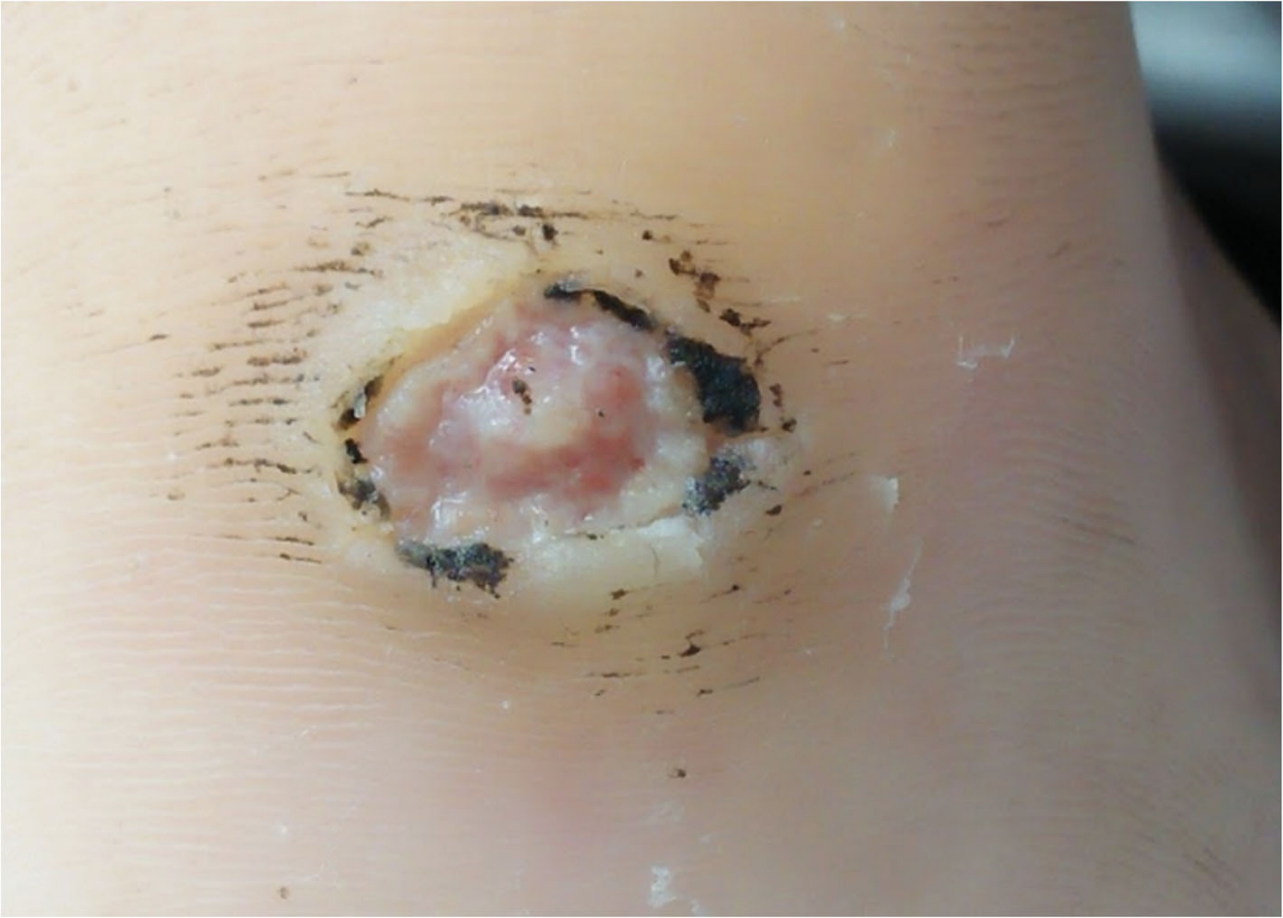
Plantar yaw: moist yellow crusted erosion overlying pink granulation tissue

These were the skin findings consistent with secondary yaws:

- Yaws papilloma: A single, large, moist, yellow-crusted nodule was noted on the skin above the upper lip (Fig. 7).
- Papulosquamous yaws lesions: Several small, flat, shiny scaly papules were noted on the arms, wrists, and buttocks with dry erosions on the elbows (Fig. 8).
- Large, hypopigmented dry patches on the thighs and legs (Figs. 8 and 9)
- Large hypopigmented patches topped with scaly papules ontop of the feet (Fig. 10).
- Plantar keratoderma and “crab yaws” were found on the soles which had multiple, dry erosions (Fig. 11) and a single large erosion with a large, moist, pink, yellow-crusted nodule (Fig. 12).

#### Case 3

This 5-year-old girl is a sibling of Case 1. The skin lesions were of an undetermined duration and were pruritic.

**Fig. 13:**
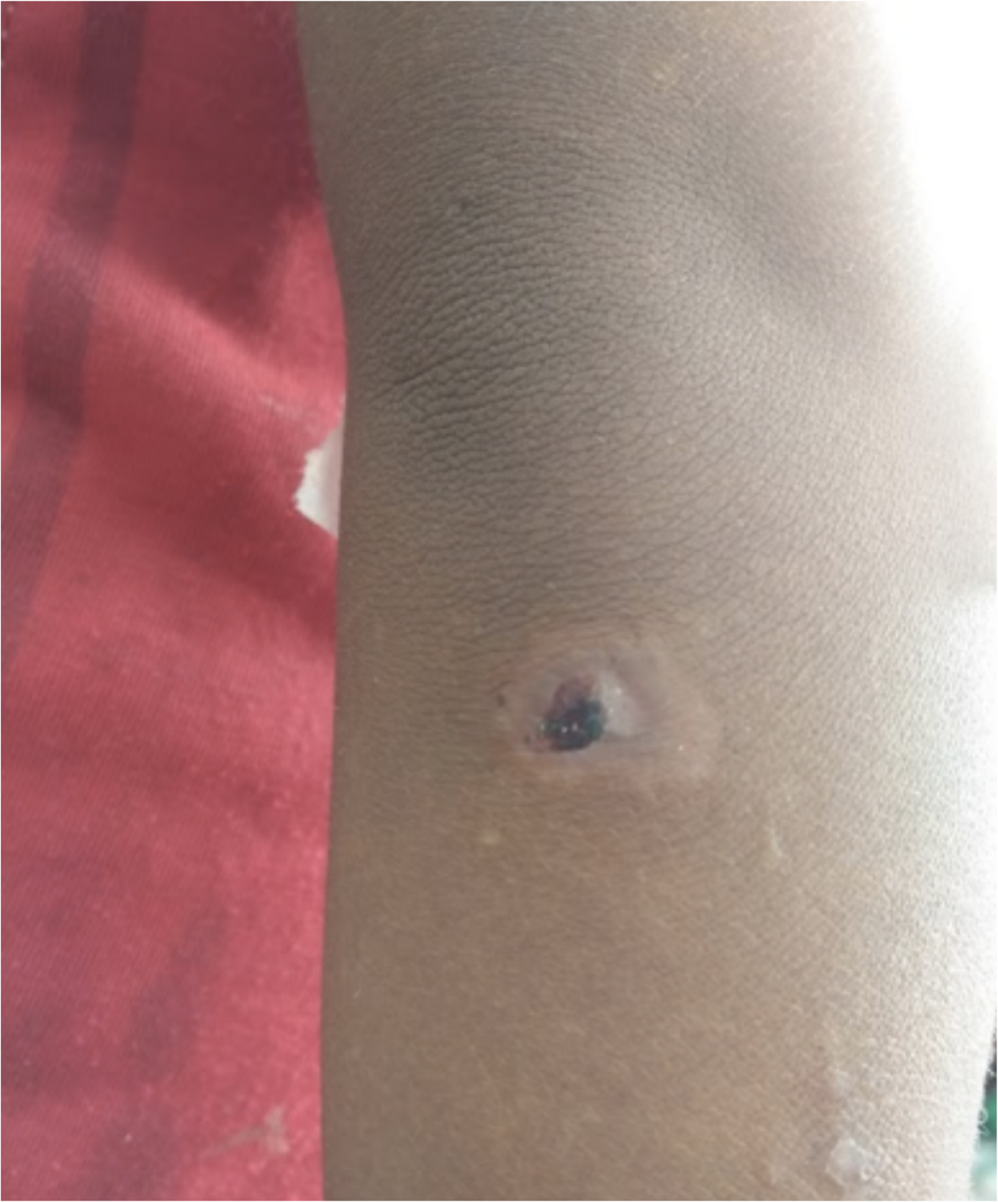
Case 3: 5-year-old girl, sibling of Case 1; black-crusted erosion and surrounding hypopigmented patch on elbow

Skin findings:

- Numerous small, whitish, scaly macules on the shoulders and arms
- Single, black-crusted erosion on the right elbow area (Fig. 13)
- Multiple, atrophic oval scars clustered on the knees

#### Case 4

The last case is a 5-year-old girl from Sultan Kudarat Province. One month prior to consult, the patient developed pruritic, recurrent, scaly papules on the dorsum of the left foot. Photographs of this patient were not taken at the time of examination.

Skin findings:

- Multiple, dry scaly papules on the dorsum of the left foot and ankle area

##### Treatment of yaws cases

All confirmed cases of yaws and their household contacts have been given one dose azithromycin by the study physicians based on WHO recommendations: (30 mg/kg body weight) for children and 2 grams for adults. Household contacts were examined for signs of yaws. All cases had seronegative non-treponemal antibody DPP tests within 1 year from treatment.

The details of the four yaws cases are summarized in Table 1.

**Table 1:** Summary of clinical and serological information on active yaws cases (*n* = 4)

STUDY SITE	AGE	SEX	DPP RESULT			TPPA TITER	INTERPRETATION	CLINICAL DIAGNOSIS	Patient Group	Skin Findings
			1	2	3					
			TREPONEMAL ANTIBODY	NON-TREPONEMAL ANTIBODY	CONTROL					
Site F SULTAN KUDARAT	5	F	Reactive	Reactive	+	-	Active & Untreated	Secondary Yaws	student	multiple dry scaly papules on dorsum of feet
Site H COTABATO	10	M	Reactive	Reactive	+	1:320	Active & Untreated	Secondary Yaws	community	large, moist, yellow crusted papillomas left axilla, yellow crusted nodules on left knee, large whitish scaly papular plaque left thigh
	9	M	Reactive	Reactive	+	1:320	Active & Untreated	Secondary Yaws; Plantar yaws	community	face: yellow crusted pink nodule upper lip area; neck - hypertrophic scar; arms - multiple scaly whitish papules; thighs - hypopigmented patches; legs- dark brown patches with nodule ontop; feet - scaly hypopigmented papular plaques; soles - dry erosions and single moist pink-yellow nodule in erosion of heel
	5	F	Reactive	Reactive	+	1:320	Active & Untreated	Secondary Yaws	community	multiple small, scaly whitish macules; black crusted erosion on elbow

*F* Female, *M* Male, *DPP* Dual Path Platform Syphilis Screen and Confirm Assay, *TPPA* Treponema Pallidum Particle Agglutination, *VDRL* Venereal Disease Research Laboratory test

#### Description of latent and past yaws cases

##### Latent yaws cases

Of the 54 adults who underwent serologic tests, eight adults (five females, three males) with ages ranging from 23 to 80 years had reactive tests to both treponemal and non-treponemal antibodies but did not have skin signs of active yaws or bone complications of yaws. Since they did not have any skin or bone signs of yaws nor syphilis, they were considered latent cases of either yaws or syphilis. They denied high risk behaviors for sexually transmitted infections and were assessed to be latent yaws cases rather than syphilis.

The latent yaws cases were found in four out of eight municipalities and in all three provinces. All except one case were household contacts of schoolchildren included in the survey. In Cotabato Province, one latent case was the mother of an active yaws patient and two latent cases were household contacts of another yaws patient. In Maguindanao, two latent cases were household contacts. In Sultan Kudarat, two latent cases were household contacts and one latent case was a community referral.

**Fig. 14:**
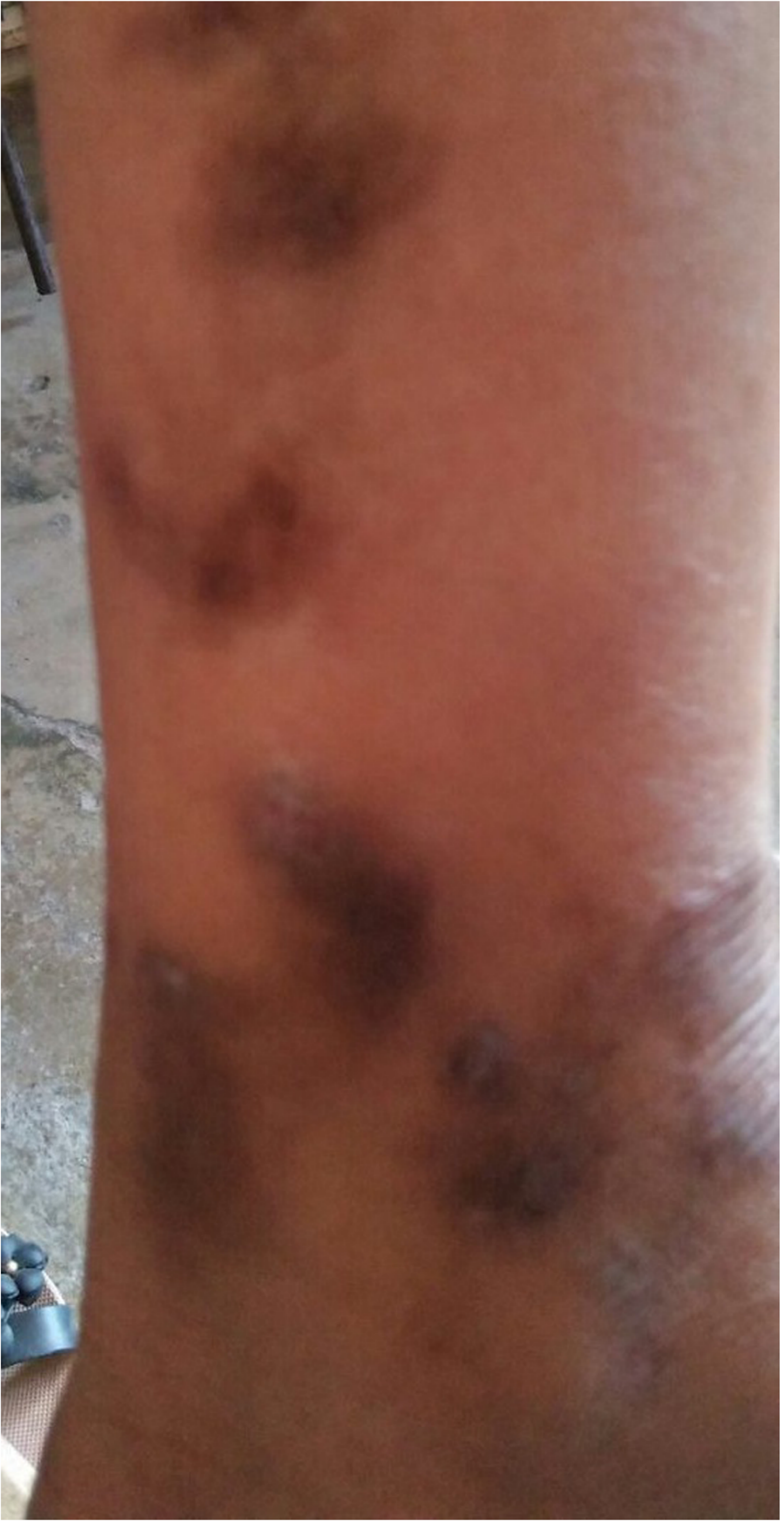
Scars of yaws lesions on the legs of a female patient with a history of yaws skin lesions in childhood and positive non-treponemal and treponemal antibodies (latent yaws)

Three of the latent cases recalled having yaws in childhood and had atrophic scars or dark brown flat scars on the legs and feet (Fig. 14). Two of them recalled having positive VDRL tests in the past. One of the male cases recalled receiving an unknown injection in the past during a medical mission. However, his TPPA titer was high 1:320. He had a pruritic, lichenified plaque on his nape at the time of examination.

##### Past yaws cases

Two adults, both 42-year old and females, had reactive tests to treponemal antibodies only. They were household members of schoolchildren from Maguindanao and Cotabato provinces. One case reported having yaws lesions during childhood and had atrophic scars on the knees upon skin examination. The second case reported having a positive VDRL test in the past but she did not have any skin signs of yaws when examined.

However, the two females did not have a history of high risk behaviors for sexually transmitted infections and lived in remote locations where yaws was endemic. They were assessed by the investigator to be more likely to be past yaws cases.

Details of the latent and past yaws cases are summarized in Table 2.

**Table 2:** Summary of clinical and serological information on latent and past yaws cases (*n* = 10)

Study site	Age	Sex	DPP result			TPPA titer				History & skin findings
			1	2	3					
			Treponemal antibody	Non-treponemal antibody	Control		Interpretation	Clinical diagnosis	Patient group	
Site A, Datu Piang, Maguindanao	27	F	Reactive	Reactive	Positive	Reactive 1:160	Active & untreated	Latent yaws	HH	Claims she had yaws during childhood; scars noted
	39	F	Reactive	Reactive	Positive	Reactive 1:320	Active & untreated	Latent yaws	HH	Claims she had yaws in childhood; dark brown patches/plaques on dorsum of feet
Site B Kabuntalan, Maguindanao	42	F	Reactive	NR	Positive	Reactive 1:320	Past/ treated	Past Yaws	HH	Claims she had yaws lesions before; atrophic scars on knees
Site F, Lambayong, Sultan Kudarat	33	M	Reactive	Reactive	Positive	Reactive 1:320	Active & untreated	Latent yaws	Community	School teacher; recalls he had yaws skin lesions in the past; (+)VDRL in 2006; given unrecalled injectable during a medical mission; no other household members with yaws lesions; No yaws skin lesions; (+) psoriasiform plaque on nape
	36	F	Reactive	Reactive	Positive		Active & untreated	Latent yaws	HH	Mother of yaws case; No skin lesions noted
	40	M	Reactive	Weakly Reactive	Positive		Active & untreated	Latent yaws	HH	Father of yaws case; No skin lesions note
Site H Tulunan, Cotabato	32	F	Reactive	Reactive	Positive	Reactive 1:320	Active & untreated	Latent yaws	Community	Recurrent pruritic papules on arms and legs after working in the fields; No yaws skin lesions; Dx. post-inflammatory hyperpigmentation due to allergic contact dermatitis
Site J Pigkawayan, Cotabato	23	F	Weakly reactive	Reactive	Positive	Reactive 1:320	Active & untreated	Latent yaws	HH	(+)VDRL in the past; aunt of student
	42	F	Weakly reactive	NR	Positive	Reactive 1:320	Past/treated	Past Yaws	HH	(+)VDRL in the past; no skin lesions noted
	80	M	Reactive	Reactive	Positive	Reactive 1:160	Active & untreated	Latent yaws	HH	No skin lesions noted

*F* Female, *M* Male, *NR* Non-reactive, *HH* Household. *DPP* Dual Path Platform Syphilis Screen and Confirm Assay, *TPPA* Treponema Pallidum Particle Agglutination, *VDRL* Venereal Disease Research Laboratory test.

**Fig. 15:**
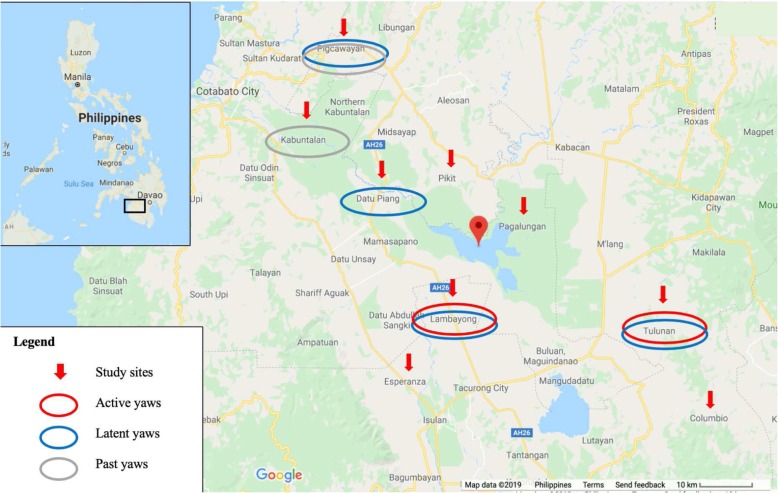
Location map of municipality study sites and yaws cases detected in Mindanao Island, the Philippines (~ 250 km horizontally)

Figure 15 shows the location of study sites and yaws cases detected in Mindanao Island, the Philippines [5, 6].

## Discussion

Yaws in the Philippines was recorded by the Bureau of Health in 1907 among leprosy patients from various parts of the country who were transferred to the Culion Leprosarium. Yaws was historically found in most parts of the Philippines, affecting 10–30% of the population in certain provinces, with most cases noted among non-Christian Filipinos in the Mindanao region (Southern Philippines) [7].

At the start of the yaws eradication campaign in 1951, the population prevalence rate was 9.6%. The yaws control programme focused on high endemic provinces such as Leyte, Samar, and selected Southern Philippines provinces and later became integrated with the rural health unit services nationwide by 1954. VDRL serologic tests (qualitative and quantitative) were used pre- and post-treatment. One to two injections of procaine penicillin in oil with 2% aluminum stearate (PAM) were administered to cases and household contacts. The yaws prevalence fell to 0.4% by December 31, 1960 [8]. In 1962–1963, the Philippine Department of Health and the WHO Treponematoses Advisory Team conducted a clinicoseroprevalence survey of selected provinces (i.e. Leyte, Samar, Cotabato, Panay Island) to assess the results of the yaws control programme. Of the 16 024 participants clinically examined, 3.3% had signs of yaws, 14.4% were reactive to VDRL, and 11.5% were reactive to Fluorescent Treponemal Antibody tests. Investigators concluded that the incidence of yaws showed a steady and continuous decline since 1922 but recommended that the health authorities should not be complacent and, instead, strongly support and continue the yaws eradication campaign [9].

The Philippine Health Statistics reported 3864 yaws cases (13.5/100 000 population) in 1961 [10]. The last official report of yaws in the Philippines recorded 424 cases (1.1/100 000 population) in 1973 [11]. The provinces included in our study have had high numbers of yaws cases recorded by the Bureau of Health since the 1950s eradication campaign and even in the subsequent Philippine Health Statistics reports.

This is the first report fully documenting yaws cases in the Philippines since 1973. A search of the published literature did not reveal any report of yaws in the Philippines since the Philippine Health Statistics report in 1973. The absence of published reports does not mean that there were no cases observed since completion of the 1950s eradication campaign. The Philippine Health Statistics included yaws as a notifiable disease but only up to 1973, after which, yaws apparently ceased to be a notifiable disease in the country.

Yaws had been reportedly common in the Bangsomoro Autonomous Region of Muslim Mindanao (BARMM) based on a survey conducted in 1999–2000 in the Liguasan Marsh area. An unpublished community skin survey was conducted by Dofitas covering five randomly selected towns of Datu Piang Municipality, Maguindanao Province, Mindanao. The skin survey detected several cases of yaws, inclusive of active cases with typical yellow crusted papillomas and those with residual scars and dyspigmentation. Among the 698 persons examined in the barangays, 82 (11.7%) were diagnosed with yaws manifesting mainly with secondary stage lesions, scars and plantar keratodermas. The majority of these yaws cases (85.4%) were children below 10 years of age. Diagnosis was based on clinical appearance and serologic tests were not performed during this survey [12]. In 2012, Dr. Sherjan Kalim surveyed women of reproductive age, their husband or sexual partner, and one of their children. Blood samples of all Standard Diagnostics Bioline Syphilis 3.0 Rapid Diagnostic Test (SD Bioline Syphilis RDT) reactive subjects were confirmed using TPPA at the Cotabato Regional and Medical Center. This unpublished study screened 1301 women and household members from Maguindanao and Cotabato provinces. Among the participants, 13.2% tested positive for *Treponema pallidum* with SD Bioline Syphilis RDT. The high seroreactivity was attributed more to yaws rather than to syphilis [12].

In 2009, Médecins Sans Frontières Switzerland (MSF) found 25.5% of women undergoing antenatal and post-natal care were reactive to *Treponema pallidum* screening tests. A local health official admitted that the cause may be yaws, a treponemal infection known to be widespread in the region [13]. The investigators had heard anecdotal reports from medical colleagues of suspected yaws cases seen in Manila and Mindanao as early as the 1970s but these were undocumented. Table 3 summarizes the reports of yaws cases (active, latent, past) based on either clinical or serological findings from past community surveys in Mindanao.

**Table 3:** Summary of yaws cases (suspected and confirmed) reported after 1973

Year	Children	> 15 years old	Total	Location	Method of detection	Reported by	Reference
2017	4	10	14	Mindanao	Clinical & serological survey (DPP & TPPA)	Dofitas B	Dofitas B, 2017 (current publication)
2012	2	97 women	99	Mindanao	Serological survey (women: RDT & VDRL quanti; children RDT)	Kalim S	Kalim S, 2013 (Unpublished)
2009	0	25.5% pregnant women	25.5% pregnant women	Mindanao	Serological survey (pre-natal/post-natal screening for syphilis)	Medicins Sans Frontieres	11
2000	77	41	118	Mindanao	Clinical skin survey	Dofitas B	Dofitas B (Unpublished)

*DPP* Dual Path Platform Syphilis Screen and Confirm Assay, *TPPA Treponema Pallidum* Particle Agglutination, *RDT* SD Bioline Syphilis Rapid Diagnostic Test, *VDRL* Venereal Disease Research Laboratory test.

The survey of Dofitas and the current study found a clustering of yaws cases in communities situated at the Liguasan Marsh, the largest swamp and marsh area in Mindanao. Apparently, the occurrence of yaws had long been known by the locals and even some health personnel in Maguindanao. The Maguindanaoan populace in the project sites have a specific term for yaws, *bakataw*, whereas other ethnolinguistic groups living there do not recall any local term for yaws.

In this study, we found strong evidence that yaws was not eliminated in the Philippines and has been in existence in the Liguasan Marsh, Mindanao for as long as the adult residents can remember. This study has provided the basis for adding the Philippines to the list of countries that are endemic for yaws, although yaws is currently proven to be limited to the study sites in Mindanao.

The major finding of this study is the confirmation of active and latent yaws in five out of the nine municipalities in provinces where Liguasan Marsh is located. The WHO criteria states that one clinically and serologically confirmed case of yaws makes the community endemic. Such is the criterion because the aim is eradication of yaws i.e. zero cases [4].

Although only two municipalities had confirmed active secondary yaws cases (i.e. four pediatric cases in Sultan Kudarat and Cotabato provinces), five municipalities had eight latent yaws cases among adult household contacts and community members. There may have been a few active yaws cases detected in this study, however, the risk of transmission is still high especially because of the lack of awareness about yaws as a health problem by local and national health authorities.

There is a possibility of latent syphilis infection among adults and older adolescents but the diagnosis of latent yaws is more likely due to the historical and current presence of yaws in the area and the low-risk profile for sexually transmitted infections of these affected individuals.

Despite the local awareness of yaws in the Liguasan Marsh areas, there have been no public health efforts to control the disease. Yaws may have persisted in the Philippines because the national campaign in the 1950s was successful in reducing the prevalence to near eradication levels, however, relatively low priority was given to sustaining the integrated yaws control measures in the local health services. Yaws stopped being a notifiable disease in the Philippines after 1973. This situation led to the persistence of yaws in communities where health care and standards of hygiene remained low, such as remote villages or areas of armed conflict [14].

The World Health Organization reports that the status of yaws is currently known in only 15 countries, with Liberia and the Philippines as the most recent additions to the list of endemic countries. The status of 76 previously endemic countries and territories remains unknown. Colombia, Ecuador, and Haiti have recently reported suspected yaws cases [15]. These countries with unknown yaws status may very well be in a similar situation as the Philippines where yaws is not recognized by health care providers anymore and is therefore unreported.

This case series provides valuable information and a guide on the clinical appearance of active yaws among Filipino children and the serological tests needed to confirm the diagnosis in a country where yaws has been a virtually forgotten disease. Health practitioners who used to recognize yaws are not present anymore. The documentation of adult latent yaws cases also raises awareness among health care providers that patients with reactive treponemal antibodies and non-treponemal antibodies may not have syphilis but may actually have latent yaws infections acquired during childhood in yaws-endemic communities.

The small number of study sites limited our search for yaws and led to underreporting of yaws cases. The WHO recommended diagnostic approach and criteria for confirmation of active yaws cases were used in this study: clinical skin signs compatible with yaws combined with serological confirmation of *Treponema pallidum* antibodies and non-*Treponema pallidum* antibodies. Ideally, polymerase chain reaction (PCR) to confirm *Treponema pallidum pertenue* would have to be performed in order to confirm the etiologic agent of yaws-like skin ulcers among children since these may also be caused by *Haemophilus ducreyi* [16] . The study did not include PCR confirmation of the etiologic agent of the skin lesions found among the four children with papillomas. None of the detected cases had skin ulcers. PCR was not performed during this study also due to the lack of PCR facilities within the Philippines and the limited budget for the conduct of the yaws study. The investigators are currently planning a collaboration with an international laboratory for future confirmation studies of yaws cases in the Philippines.

## Conclusions

The clinical, serological, and histopathological confirmation of four yaws cases among children prove that this disease is endemic in at least two out of nine municipalities of the Liguasan Marsh, Mindanao. The Philippines is now the 14th country endemic for yaws. This documentation of confirmed yaws in the Philippines has provided the necessary evidence and stimulus to develop a yaws control and eradication program as one of the country’s neglected tropical diseases. With the renewed knowledge about yaws and its public health importance, the national and local health authorities have been continuing the search for more hidden yaws cases and have detected additional active and latent yaws. An ongoing surveillance of yaws cases is being conducted in the rest of the country. Yaws should be taught to health personnel and communities at risk. The current WHO yaws eradication strategy entails mapping, surveillance, strengthening primary health care services, Targeted or Total Community Treatment with one oral dose of azithromycin and encourages integration with other neglected tropical diseases programs [4].

## Data Availability

The data that support the findings of this study are available from Philippine Department of Health’s Health Systems Research Management but restrictions apply to the availability of these data, which were used under license for the current study, and so are not publicly available. Data are however available from the authors upon reasonable request and with permission of the Philippine Department of Health’s Health Systems Research Management.

## Abbreviations

BARMM: Bangsamoro Autonomous Region of Muslim Mindanao
DPP: Dual path platform syphilis screen and confirm assay
MSF: Médecins Sans Frontières Switzerland
PCR: Polymerase chain reaction
RDT: SD Bioline Syphilis Rapid Diagnostic Test
TPPA: *Treponema Pallidum* Particle Agglutination
VDRL: Venereal disease research laboratory test
WHO: World Health Organization

## References

[1] Bravo FG TC, Ezzedine K. Endemic nonvenereal treponematosis. In: Fitzpatrick’s Dermatology. Volume 1, 9th edn. Edited by Kang Sea. New York: McGraw-Hill Education; 2019. p. 3177-8.

[2] Nagreh DS (1986). Yaws. CUTIS Trop Dermatol.

[3] WHO (2012). Summary report of a consultation on the eradication of yaws.

[4] WHO (2018). Eradication of Yaws-- A Guide for Programme Managers.

[5] Google: Map of Liguasan Marsh, Mindanao. In: Google Maps. Google; 2019: Map of Mindanao Island and Liguasan Marsh. Accessed 11 Nov 2019.

[6] Google (2019). Map of the Philippines. *Google Maps.* Google: Map of the Philippines.

[7] Cruz AH (1953). Integration of yaws control into the permanent health structure of the Philippines. Bull World Health Organ.

[8] Cruz A, Suva J, Justiniano G (1961). The yaws control programme in the Phiippines with WHO/UNICEF assistance. J Philipp Med Assoc.

[9] Cruz AH, Justiniano GS, Camena TC (1965). A critical evaluation of yaws in the Philippines. J Philipp Med Assoc.

[10] Philippine Health Statistics 1961 (1961). Health Bo. Philippines.

[11] Philippine Health Statistics 1973 (1973). Health Bo. Philippines.

[12] WHO (2018). Report of a global meeting on yaws eradication surveillance, monitoring and evaluation, Geneva, 29–30 January 2018.

[13] Philippines (2009). High rate of treponematosis among pregnant IDP women. *The New Humanitarian*.

[14] Meheus A, Antal GM (1992). The endemic treponematoses: not yet eradicated. World Health Stat Q.

[15] Yaws [https://www.who.int/news-room/fact-sheets/detail/yaws].

[16] Mitja O, Lukeheart S, Pokowas G, Moses P, Kapa A, Godornes C (2014). *Haemophilus ducreyi* as a cause of skin ulcers in children from a yaws-endemic area of Papua New Guinea: a prospective cohort study. Lancet Glob Health.

